# Polysorbate-Based Carriers Encapsulating Oxygen-Deficient Nanoparticles for Targeted and Effective Chemo-Sonodynamic Therapy of Glioblastoma

**DOI:** 10.3390/ijms262010235

**Published:** 2025-10-21

**Authors:** Hyeon Ju Kang, Quan Truong Hoang, Nguyen Cao Nguyen, Binh Thi Thanh Pham, Thuy Giang Nguyen Cao, Vasanthan Ravichandran, Min Suk Shim

**Affiliations:** Department of Nano-Bioengineering, Incheon National University, Incheon 22012, Republic of Korea; yuj005129@inu.ac.kr (H.J.K.); thquan0401@gmail.com (Q.T.H.);

**Keywords:** sonodynamic therapy, blood–brain barrier, polysorbate, MnWOx, glioblastoma

## Abstract

Glioblastoma multiforme (GBM) is the most aggressive brain tumor with a high recurrence rate and mortality. A major obstacle to the effective treatment of GBM is the blood–brain barrier (BBB), which hinders the transfer of therapeutic cargo to the tumor lesion. Polysorbate-coated drug carriers are known to efficiently cross the BBB via apolipoprotein E (ApoE)-mediated transcytosis. In this study, we developed cancer-targeted nanocarriers using folic acid (FA)-conjugated polysorbate (Tween 80, T80) for safe and efficient chemo-sonodynamic combination therapy against GBM. T80-based nanocarriers effectively co-encapsulated doxorubicin (DOX, chemotherapeutic agent) and oxygen-deficient MnWOx nanoparticles (sonosensitizer). FA-conjugated T80 nanocarriers encapsulating DOX and MnWOx (FA-T-DOX@MnWOx) boosted the cellular uptake of DOX in human glioblastoma U87MG cells. The efficient ability of the T80-based drug carriers to cross the BBB was demonstrated using an in vitro transwell BBB model. In addition, sonosensitizer MnWOx nanoparticles in the T80-based carriers triggered GSH depletion, synergistically enhancing intracellular reactive oxygen species (ROS) generation in U87MG cells upon US irradiation. As a result, FA-T-DOX@MnWOx combined with US triggered significant apoptosis in U87MG cells. This study demonstrated that FA-conjugated, MnWOx-loaded, T80-based nanocarriers capable of crossing the BBB hold significant potential for treating GBM through a combined chemo-sonodynamic therapy.

## 1. Introduction

Glioblastoma multiforme (GBM) is recognized as the most aggressive and lethal primary brain tumor [1]. The current standard therapy for GBM, which combines surgical resection, radiotherapy, and chemotherapy, faces a significant challenge due to its side effects on normal tissues and tumor recurrence [2]. Chemotherapy, when combined with other therapies, including immunotherapy, photothermal therapy (PTT), and photodynamic therapy (PDT), has shown synergistic effects against GBM while minimizing side effects on normal tissues [3,4,5]. However, these combination therapies still face several challenges, including the low cancer-specificity of drugs and their limited penetration into brain tissues [6,7].

Sonodynamic therapy (SDT), which employs sonosensitizers and ultrasound (US) to produce reactive oxygen species (ROS), is a noninvasive treatment option for cancer treatment [8,9]. SDT has received significant attention as an effective option for treating tumors located deep within the body, primarily due to the deeper tissue penetration by US compared to light used in phototherapy (e.g., PTT and PDT) [10,11]. Recently, SDT has gained great attention as a promising treatment option for GBM treatment because it can selectively and noninvasively kill tumor cells while minimizing toxicity to surrounding healthy cells [9,12]. Due to the high dependence of SDT efficacy on the potency of sonosensitizers, it is crucial to develop safe sonosensitizers that exhibit high tumor-targeting capabilities and low quantum yields of ROS production for effective SDT [13,14].

Oxygen-deficient bimetallic oxide semiconductors have recently become effective sonosensitizers due to their ability to improve ROS quantum yields by suppressing electron-hole recombination [15,16]. We previously demonstrated that oxygen-deficient MnWOx nanoparticles (NPs) efficiently generate ROS upon US exposure [17]. Moreover, the manganese component in the MnWOx NPs was shown to induce glutathione (GSH) depletion in cancer cells [15,17]. This depletion led to an increase in intracellular ROS, thereby enhancing the efficacy of SDT in cancer cells [15,17]. Despite their high potency in SDT, the clinical application of MnWOx NPs is significantly hindered by their poor aqueous solubility and limited tumor-specificity [17].

A key challenge to SDT in GBM treatment is the effective delivery of sonosensitizers to the tumor lesion, owing to the presence of blood–brain barrier (BBB) [12,18]. Therefore, the development of sonosensitizers that can efficiently cross the BBB is highly required for successful SDT in GBM treatment. In recent years, nanocarriers have demonstrated great promise as an effective approach to enhance the intracellular delivery, bioavailability, and tumor-targeting ability of sonosensitizers [19,20]. Various nanocarriers have been developed to transport their payloads across the BBB via receptor-mediated transcytosis [7,21]. Drug carriers coated with polysorbate 80 (Tween 80, T80), a nonionic surfactant, have demonstrated enhanced drug delivery across the BBB [22,23,24]. T80 binds with plasma lipoprotein, apolipoprotein E (ApoE), which subsequently attaches to low-density lipoprotein receptors on brain endothelial cells [24,25]. This interaction allows T80-based drug carriers to facilitate drug delivery to the brain via receptor-mediated transcytosis [25].

**Figure 1:**
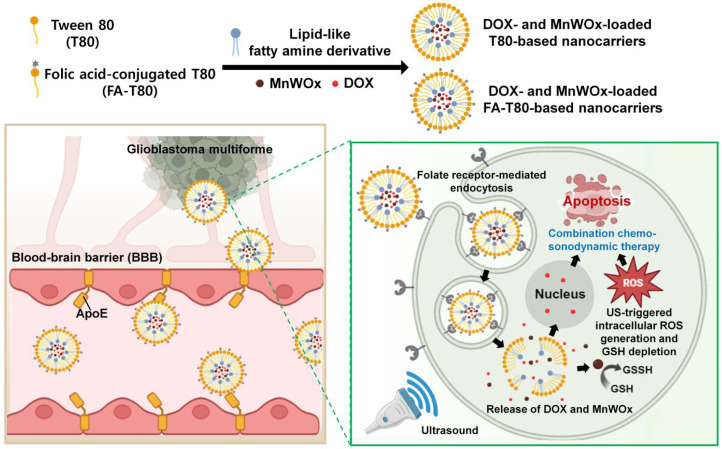
Schematic illustration of the combined chemo-sonodynamic therapy using folic acid (FA)-conjugated Tween 80 (T80) drug carriers encapsulating doxorubicin (DOX) and MnWOx nanoparticles (FA-T-DOX@MnWOx NPs). The T80-based drug carriers can cross the blood–brain barrier (BBB) via ApoE-mediated transcytosis and are subsequently taken up by glioblastoma cells through folate receptor-mediated endocytosis. Upon internalization, the T80-based drug carriers release DOX and MnWOx NPs. Ultrasound (US) irradiation then triggers the MnWOx NPs to generate reactive oxygen species (ROS). The MnWOx NPs also deplete glutathione (GSH), which synergistically boosts ROS levels in the glioblastoma cells. The released DOX also triggers apoptosis, leading to a synergistic increase in cell death.

In this study, we fabricated safe and cancer-targeted T80-based nanocarriers encapsulating MnWOx NPs for the treatment of GBM by facilitating BBB penetration (Figure 1). We also loaded the chemotherapeutic agent doxorubicin (DOX) into the T80-based nanocarriers to enable combination chemo-SDT. Moreover, T80 was conjugated with folic acid (FA) for tumor-targeted SDT combined with chemotherapy. The FA-conjugated T80-based nanocarriers can be efficiently internalized by GBM cells that overexpress folate receptors [26,27]. FA-modified T80-based nanocarriers, encapsulating both DOX and MnWOx NPs (FA-T-DOX@MnWOx) are hypothesized to be effective for chemo-SDT against GBM. We anticipate that GSH-depleting MnWOx NPs in T80-based drug carriers can effectively cross the BBB and subsequently trigger the generation of ROS within GBM cells upon ultrasound (US) treatment. The physicochemical properties of various T80-based drug carriers were evaluated, and their chemo-SDT activity against GBM was assessed in vitro.

## 2. Results and Discussions

### 2.1. Fabrication and Characterization of MnWOx NPs

Oxygen vacancy-defected bimetal oxide NPs are promising sonosensitizers owing to their high yield of ROS production upon US exposure [15,16]. The oxygen vacancies within their structure act as electron-trapping sites, effectively suppressing electron–hole recombination. We previously reported that MnWOx NPs with oxygen vacancies efficiently produce ROS under US irradiation [17]. In this study, hydrophobic MnWOx NPs and DOX were co-loaded into cancer-specific FA-bearing T80-based formulations for tumor-targeted chemo-SDT against GBM with increased bioavailability.

**Figure 2:**
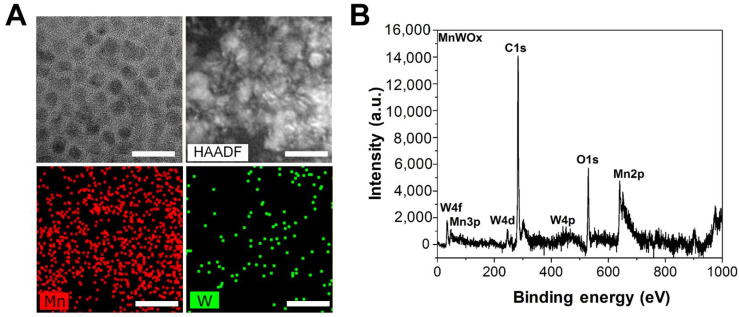
(**A**) TEM and high-angle annular dark-field scanning TEM (HAADF-STEM) images of MnWOx NPs. Scale bars indicate 30 nm. (**B**) High-resolution XPS spectra of MnWOx NPs.

We fabricated MnWOx NPs with W(CO)_6_ and manganese acetylacetonate [Mn(acac)_3_] as precursors using an organic-phase one-pot synthesis, as previously reported [17]. Transmission electron microscopy (TEM) images revealed that the MnWOx NPs had elongated grains with an average diameter ranging from 6 to 8 nm (Figure 2A). The presence of Mn and W elements in the MnWOx NPs was verified by energy-dispersive X-ray spectroscopy (EDS) elemental mapping (Figure 2A). X-ray photoelectron spectroscopy (XPS) was used to characterize the compositions and oxidation states of MnWOx NPs (Figure 2B and Figure S1). The binding energy peaks at 285 eV and 530 eV in the XPS spectrum correspond to C 1s and O 1s, respectively (Figure 2B). In addition, the peaks at 640 eV and 652 eV verified the presence of Mn^2+^, corresponding to the binding energies of Mn^2+^(2p _3/2_) and Mn^2+^(2p _1/2_), respectively (Figure S1A). The peaks for W^5+^(4f _5/2_) and W^6+^(4f _7/2_) appeared at 35.5 and 33.5 eV, respectively (Figure S1A). XPS analysis determined the value of “x” in the MnWOx NPs to be approximately 2.8. These XPS results demonstrate that MnWOx NPs were successfully fabricated, which is consistent with the results from a previous study [17].

### 2.2. Synthesis and Characterization of FA-Conjugated Tween 80

**Figure 3:**
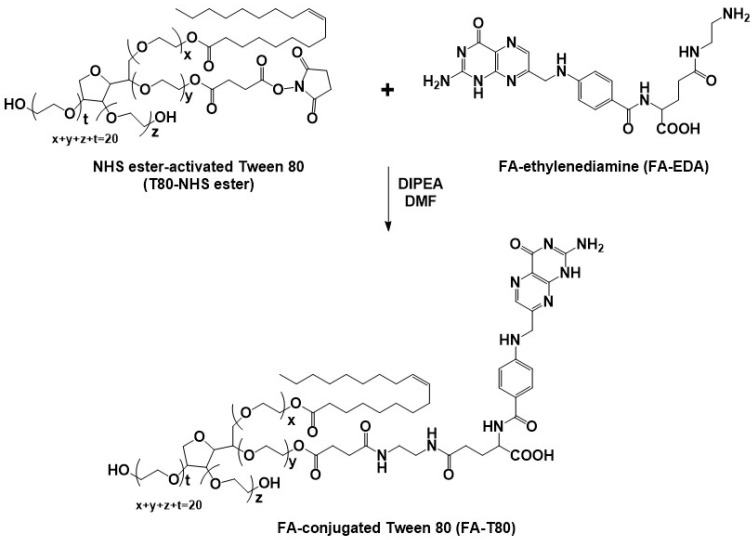
Synthetic scheme of FA-conjugated Tween 80.

Cancer-targeting FA-conjugated T80 (FA-T80) was prepared by reacting NHS ester-activated T80 (T80-NHS ester) and amine-functionalized FA-ethylenediamine (FA-EDA) (Figure 3). The hydroxyl groups of T80 were converted into carboxylic acid groups using succinic anhydride, with subsequent activation via an EDC/NHS strategy to prepare T80-NHS ester (Figure 3 and Figure S2). The synthesis of FA-EDA was accomplished by coupling FA with *N*-Boc-ethylenediamine, with subsequent deprotection of the Boc groups (Figure S2). The detailed syntheses of T80-NHS ester and FA-EDA are described in the Supplementary Materials.

The chemical structures of T80-NHS ester, FA-EDA, and FA-T80 were confirmed by ^1^H NMR (Figure S3–S5 in the Supplementary Materials). The proton signals for the poly(ethylene oxide) of T80-NHS ester were observed at 3.65 ppm (Figure S3). The peak at 0.88 ppm is attributed to the terminal methyl protons of the oleic acid side chains in T80 (Figure S3). T80-NHS ester showed a distinct and sharp peak around 2.8 ppm, which is the characteristic signal for the four equivalent protons of the NHS ring (Figure S3). The successful synthesis of FA-EDA was also confirmed by ^1^H NMR. The spectrum of FA-EDA was characterized by the methylene protons of ethylenediamine, which appeared at 2.7 ~ 2.9 ppm (Figure S4). The peaks at 6.6 and 7.6 ppm are attributed to the aromatic protons of the *p*-aminobenzoyl ring in FA (Figure S4). The synthesis of FA-T80 was confirmed by ^1^H NMR, where methylene protons (δ 3.65 ppm) in the poly(ethylene oxide) (PEO) and aromatic protons (δ 6.6, 7.6, and 8.7 ppm) in the FA were observed (Figure S5).

### 2.3. Fabrication and Characterization of MnWOx NP-Loaded T80 Nanocarriers

T80-based nanocarriers encapsulating MnWOx NPs and/or DOX were prepared via self-assembly. Hydrophobic long alkyl chains and hydrophilic PEO chains of T80 allow self-assembly. To enhance the encapsulation efficiency of the hydrophobic MnWOx NPs, a lipid-like fatty amine derivative (FAD) was synthesized and added to the formulation. The detailed synthesis of FAD is described in the Supplementary Materials (Figure S6).

**Figure 4:**
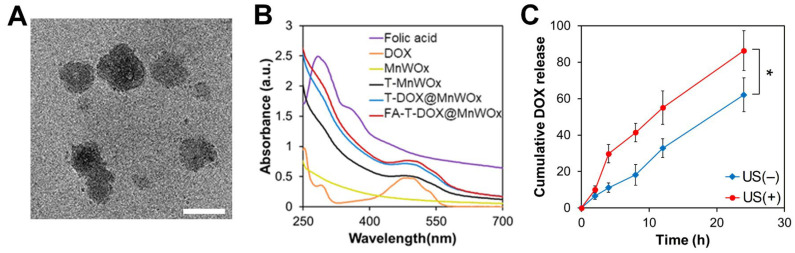
(**A**) TEM image of FA-T-DOX@MnWOx nanocarriers. Scale bar is 100 nm. (**B**) Absorbance spectra of various samples. (C) DOX release profiles from FA-T-DOX@MnWOx in PBS before and after US irradiation (1 MHz, 0.5 W/cm^2^, 2 min) at different incubation times (*n* = 3). Data are presented as mean ± SD (* *p* < 0.05).

The formulation optimization of MnWOx-loaded T80 nanocarriers was performed by varying the T80-to-FAD (*w*/*w*) ratios. After careful optimization, the 10:2 (*w*/*w*) ratio of T80-to-FAD was found to be the most effective in achieving the smallest particle size of MnWOx-loaded T80 nanocarriers (Table S1 in the Supplementary Materials). This ratio resulted in a particle size of 214.6 nm [measured by dynamic light scattering (DLS)] and was used to prepare T-DOX@MnWOx and FA-T-DOX@MnWOx. T-DOX@MnWOx and FA-T-DOX@MnWOx nanocarriers had average hydrodynamic sizes of 201.4 nm and 221.9 nm, respectively, as measured by DLS. Their surface charges were nearly neutral (–0.13 ± 0.28 and 1.22 ± 0.05 mV, respectively), indicating the nonionic nature of T80. TEM images of the FA-T-DOX@MnWOx confirmed their spherical shape and showed diameters ranging from 140 to 230 nm (Figure 4A). The successful encapsulation of DOX within the T80-based carriers was further confirmed by UV–Vis spectroscopy. As shown in Figure 4B, the absorption spectra of FA, DOX, MnWOx, T-MnWOx, T-DOX@MnWOx, and FA-T-DOX@MnWOx were compared. A characteristic absorption peak of DOX was observed at 484 nm, verifying the successful incorporation of DOX into the T80-based carriers. The DOX encapsulation efficiency of FA-T-DOX@MnWOx was calculated to be 16.7%.

### 2.4. Enhanced DOX Release Profile from FA-T-DOX@MnWOx

To demonstrate the US-activated drug release from FA-T-DOX@MnWOx, the amount of DOX released from FA-T-DOX@MnWOx incubated in PBS (pH 7.4, 37 °C) was quantified before and after exposure to US irradiation. Regardless of the US irradiation, the cumulative amount of DOX increased gradually within 24 h (Figure 4C). In contrast, the cumulative amount of DOX released from FA-T-DOX@MnWOx dramatically increased when it was pretreated with US (0.5 W/cm^2^, 2 min). Under pre-treatment with US, FA-T-DOX@MnWOx released 86.3% of DOX within 24 h, compared to only 62.1% without US exposure (Figure 4C). This result verifies the US-activated drug release by MnWOx NP-loaded T80 carriers. The US-activated drug release can be explained by the acoustic cavitation effect of US, which facilitates drug diffusion.

### 2.5. Cancer-Specific Cellular Uptake Analysis of FA-T-DOX@MnWOx

**Figure 5:**
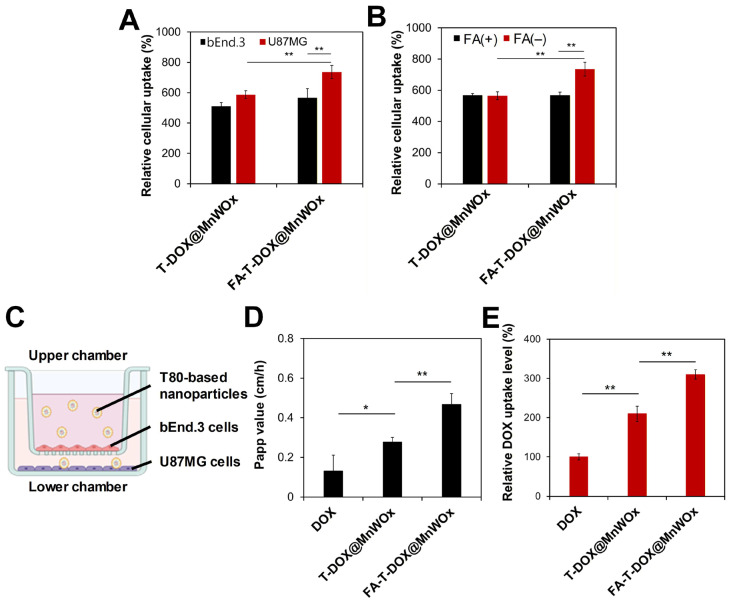
(**A**) Relative uptake levels of T-DOX@MnWOx, and FA-T-DOX@MnWOx in U87MG and bEnd.3 cells. (**B**) Relative uptake levels of T-DOX@MnWOx and FA-T-DOX@MnWOx following pre-treatment with FA in U87MG cells. (**C**) Schematic diagram of in vitro BBB model. (**D**) Apparent permeability coefficient (P_app_) of DOX, T-DOX@MnWOx, and FA-T-DOX@MnWOx measured using an in vitro BBB model. (**E**) Relative DOX uptake of FA-T-DOX@MnWOx by U87MG cells after crossing BBB layers (*n* = 3). Data are presented as mean ± SD (* *p* < 0.05; ** *p* < 0.01).

U87MG cell line, established from a human glioblastoma, was used for the in vitro evaluation of various T80-based carriers. The U87MG cell line is one of the most widely employed cellular models in cancer research [28]. Folate receptors are overexpressed on the plasma membranes of U87MG cells [27]. To investigate whether FA-conjugated T80-based drug carriers can achieve cancer-specific drug delivery, T-DOX@MnWOx and FA-T-DOX@MnWOx were incubated with U87MG cells. As shown in Figure 5A, FA-T-DOX@MnWOx was more effectively taken up by the folate receptor-overproducing U87MG cells than FA-free T-DOX@MnWOx. This result indicates that FA-T-DOX@MnWOx was more readily taken up by U87MG cells. To assess the effect of FA on receptor-mediated endocytosis of FA-T-DOX@MnWOx, we conducted a competition binding study where U87MG cells were pre-incubated with free FA before the addition of T-DOX@MnWOx and FA-T-DOX@MnWOx. The pre-incubation with free FA substantially decreased the internalization of FA-T-DOX@MnWOx by U87MG cells (Figure 5B). In contrast, the cellular internalization of the FA-free T-DOX@MnWOx by U87MG cells was not significantly compromised by the pre-incubation with FA. These results suggest that folate receptor-mediated endocytosis is responsible for the increased uptake of FA-T-DOX@MnWOx by U87MG cells.

### 2.6. Efficient BBB Transcytosis of MnWOx-Loaded T80-Based Carriers

Efficient delivery of anticancer agents across the BBB to brain tumors is a critical step for the successful treatment of gliomas. T80-based nanocarriers are known to cross the BBB by adsorbing ApoE, which then binds to lipoprotein receptors on the BBB’s luminal surface. This process facilitates receptor-mediated transcytosis, enabling the NPs to cross the barrier [22,24]. To investigate whether FA-T-DOX@MnWOx can effectively cross the BBB and reach glioblastoma, we established an in vitro transwell BBB model. The simulated BBB bilayer was constructed by culturing bEnd.3 brain endothelial cells in the upper chamber and human glioblastoma U87MG cells in the lower chamber (Figure 5C). A porous membrane was used to separate the upper and lower chambers. bEnd.3 cells in the upper chamber were treated with free DOX, T-DOX@MnWOx, or FA-T-DOX@MnWOx, and the amount of DOX transported to the lower chamber was quantified. As shown in Figure 5D, the encapsulation of DOX into T80-based nanocarriers significantly increased its transport across the BBB, as evidenced by the higher apparent permeability coefficient (P_app_) values observed for the nanocarriers compared to free DOX. Notably, FA-T-DOX@MnWOx exhibited the highest P_app_ value, demonstrating its efficient BBB penetration. It was reported that FA can enhance the BBB penetration by binding to specific transporters or receptors on brain endothelial cells, including folate receptors, reduced folate carrier, and proton-coupled folate transporter [29].

The intracellular uptake levels of free DOX and MnWOx-loaded T80 carriers by U87MG cells were quantified to evaluate the capability of FA-conjugated, T80-based nanocarriers to cross the BBB and target brain tumors. These uptake levels were measured after 4 h of incubation with bEnd.3 cells in the upper chambers. As presented in Figure 5E, T-DOX@MnWOx and FA-T-DOX@MnWOx significantly improved the intracellular uptake level of DOX into U87MG cells. Moreover, FA-T-DOX@MnWOx showed increased DOX uptake in U87MG cells compared to FA-free T-DOX@MnWOx. This result demonstrates the enhanced tumor-targeting efficacy of FA-conjugated, T80-based nanocarriers. The efficient cellular uptake of FA-T-DOX@MnWOx into U87MG cells might be attributed to the favorable binding of the carriers with the cell membranes via FA-folate receptor interaction [27]. The enhanced cellular uptake of MnWOx NPs via FA-conjugated T80 allows for a reduced therapeutic dose, thereby minimizing the intrinsic cytotoxicity of the MnWOx NPs.

### 2.7. US-Activated Intracellular ROS Generation by T80 Nanocarriers Encapsulating MnWOx

**Figure 6:**
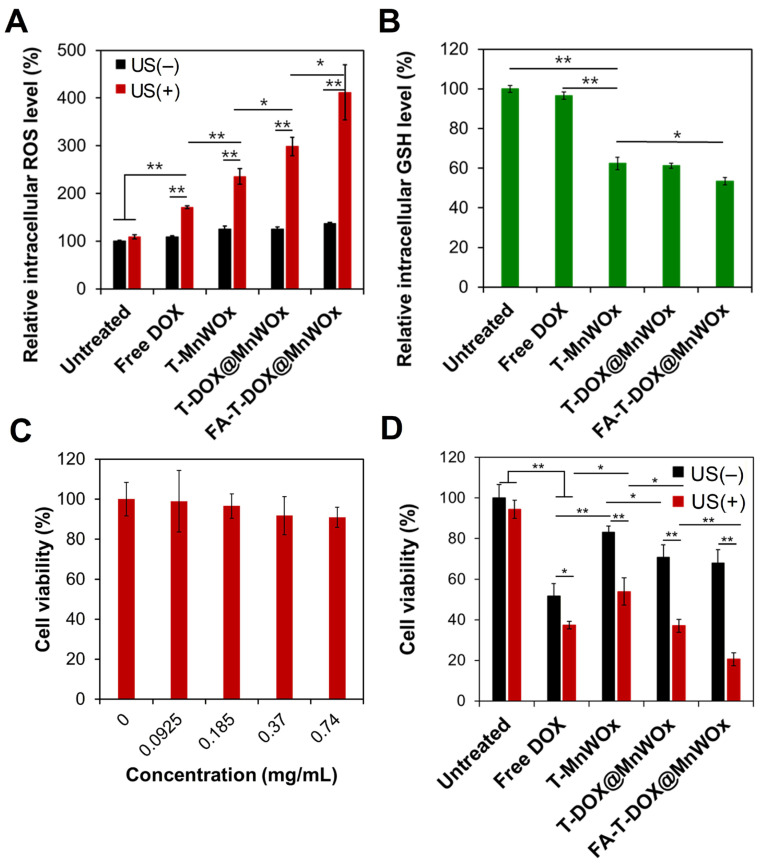
(**A**) Relative ROS levels in U87MG cells incubated with various T80-based carriers before and after US irradiation (0.5 W/cm^2^, 1 MHz, 2 min) (*n* = 3). (**B**) Intracellular GSH levels in U87MG cells after treatments with various T80-based carriers. (**C**) The viability of U87MG cells treated with empty FA-T80-based carriers at their various concentrations. (**D**) Viabilities of U87MG incubated with various T80-based carriers (2.5 μg/mL DOX, 7.5 μg/mL MnWOx, 0.37 mg/mL T80) before and after 2 min of US irradiation (1 MHz, 0.5 W/cm^2^). Data are presented as mean ± SD (* *p* < 0.05; ** *p* < 0.01).

MnWOx NPs are sonocatalytic semiconductors. Under ultrasound (US) irradiation, the energy released from the cavitation effect triggers the generation of electron-hole pairs in the MnWOx NPs [15]. This separation of charge carriers subsequently undergoes redox reactions with oxygen and water to generate ROS. To demonstrate the sonocatalytic generation of ROS by MnWOx NPs, the intracellular levels of ROS in U87MG cells treated with MnWOx-loaded T80 samples were measured before and after US irradiation. Free DOX and US significantly increased ROS levels in U87MG cells (Figure 6A), which was consistent with the result in a previous study [30]. In addition, U87MG cells treated with MnWOx-loaded T80 nanocarriers elevated intracellular ROS levels significantly after US irradiation compared to free DOX (Figure 6A). When the DOX was loaded into T80 nanocarriers (T-DOX@MnWOx), the cells generated even higher levels of ROS after US exposure. Notably, FA-T-DOX@MnWOx combined with US treatment produced the most significant increase in ROS levels in U87MG cells. This enhanced ROS generation is attributed to the increased cellular uptake of FA-T-DOX@MnWOx, which occurs through folate receptor-mediated endocytosis.

The intracellular levels of ROS in U87MG cells after treatment with various samples were also analyzed using a fluorescence microscope (Nikon Eclipse Ti-S). We observed the strongest ROS-indicating green fluorescence in the cells when treated with FA-T-DOX@MnWOx and US (Figure S7A). This fluorescence imaging result well agreed with the intracellular ROS measurement by flow cytometry (Figure 6A). These results indicate that FA-T-DOX@MnWOx is an effective sonosensitizer for U87MG cells.

### 2.8. Efficient GSH Depletion by MnWOx-Loaded T80 Nanocarriers

GSH depletion is a key factor in regulating intracellular ROS accumulation [31,32]. Our previous studies reported that MnWOx NPs effectively deplete GSH, thereby amplifying ROS production [17]. To evaluate this effect, U87MG cells were treated with different formulations and analyzed using Thiol Tracker Violet staining (Figure 6B and Figure S7B). Treatment with free DOX resulted in only a minimal decrease (3.4%) in intracellular GSH. In contrast, MnWOx-loaded T80 carriers caused a substantial reduction in the GSH levels of U87MG cells. The cancer-targeting FA-T-DOX@MnWOx was the most effective in depleting intracellular GSH in U87MG cells (Figure 6B and Figure S7B). The superior GSH depletion by FA-T-DOX@MnWOx is attributed to its efficient cellular uptake in U87MG cells via FA-folate receptor interaction (Figure 5A). The findings suggest that MnWOx-mediated GSH depletion is responsible for the increased intracellular ROS accumulation observed in U87MG cells (Figure 6A).

### 2.9. Cancer-Selective Combined Chemo-SDT Using FA-T-DOX@MnWOx

To demonstrate the low toxicity of T80-based nanocarriers, the viability of U87MG cells was measured after incubation with blank T80-based nanocarriers at various concentrations. The cells remained at least 90% viable at concentrations up to 0.74 mg/mL of T80 (Figure 6C). T80 has been reported to undergo enzymatic hydrolysis and rapid systemic elimination in the body [33]. After MnWOx NPs are released from T80-based nanocarriers, they are expected to transform into soluble Mn and W ions. These soluble metal ions can be effectively cleared from the body via renal excretion, as demonstrated previously [17].

The sonotoxicity of various MnWOx-loaded T80-based nanocarriers against U87MG cells was evaluated using a standard MTT assay. The viability of U87MG cells treated with various T80-based nanocarriers before and after US irradiation (1 MHz, 0.5 W/cm^2^, 2 min) was quantified. As shown in Figure 6D, the viability of U87MG cells was significantly reduced when they received free DOX and DOX-loaded T80-based nanocarriers. US irradiation further reduced the viability of the cells treated with MnWOx-encapsulating nanocarriers. For example, the viabilities of the cells after treatment with T-MnWOx were reduced from 83.1% to 53.9% after US irradiation (Figure 6D). The increased cytotoxicity of T-MnWOx upon US irradiation might be attributed to the US-triggered ROS generation by MnWOx. Notably, the highest sonotoxicity (20.5% cell viability) was observed when FA-T-DOX@MnWOx was added to the cells under US treatment (Figure 6D). This might be attributed to the efficient cellular uptake of FA-T-DOX@MnWOx in U87MG cells. This result highlights the high potency of combined chemo-SDT using MnWOx-loaded T80-based nanocarriers with a single treatment of US. Multiple US treatments would be clinically feasible to further enhance the therapeutic efficacy of FA-T-DOX@MnWOx.

**Figure 7:**
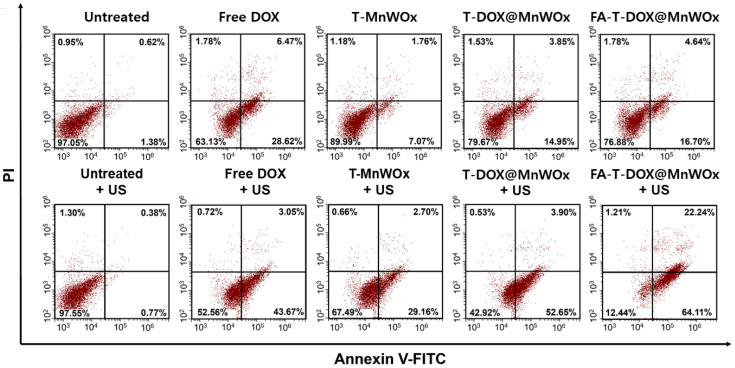
Apoptosis analysis of U87MG cells treated with various samples before and after irradiation of US (1 MHz, 0.5 W/cm^2^, 2 min).

To investigate whether sonotoxicities of MnWOx-loaded T80 nanocarriers result from apoptosis, apoptosis rates of U87MG cells treated with various MnWOx-loaded T80 nanocarriers were analyzed before and after US exposure (Figure 7). Annexin V-FITC/PI staining assay revealed that U87MG cells treated with MnWOx-loaded T80 nanocarriers caused a slight increase in the percentage of total apoptotic cells (including both early and late apoptosis) without US treatment. However, the total apoptosis rate increased significantly in cells treated with MnWOx-loaded T80 nanocarriers under US irradiation (Figure 7). The highest apoptosis rate of U87MG cells was observed for FA-T-DOX@MnWOx combined with US irradiation (Figure 7). This result might be ascribed to the highest level of intracellular ROS generation by FA-T-DOX@MnWOx upon US irradiation. The apoptosis results, along with the intracellular ROS generation analysis (Figure 6A), demonstrate that the sonodynamic effects of MnWOx-loaded T80 nanocarriers cause a rise in oxidative stress, which triggers apoptosis in GBM.

## 3. Materials and Methods

### 3.1. Materials

T80, DOX hydrochloride, 3-(4,5-dimethylthiazol-2-yl)-2,5-diphenyltetrazolium bromide (MTT), W(CO)_6_, and Mn(acac)_3_ were purchased from Sigma Aldrich (St. Louis, MO, USA). Dulbecco’s modified Eagle’s medium (DMEM) and fetal bovine serum (FBS) were supplied from Thermo Fisher Scientific (Waltham, MA, USA). b.End3 and U87MG cells were obtained from ATCC (Manassas, VA, USA). A Sonicator 740 (Mettler Electronics Corp., Anaheim, CA, USA) was utilized for US irradiation.

### 3.2. Preparation of MnWOx- and DOX-Loaded T80 Nanocarriers

MnWOx NPs were prepared using W(CO)_6_ and Mn(acac)_3_ following the same method as reported in our previous study [17]. To prepare T-MnWOx, a 10% (*w*/*v*) T80 solution was prepared in PBS. MnWOx NPs (1 mg) were dispersed in 100 µL of dichloromethane (DCM). To enhance the encapsulation efficiency of the hydrophobic MnWOx NPs, lipid-like fatty amine derivative (FAD) was prepared as reported in our previous study [34] (Supplementary Materials). FAD dissolved in DCM (100 µL) was mixed with the MnWOx suspension and subsequently added to a 100 µL T80 solution/900 µL PBS mixture. The resulting mixture was sonicated for 15 min using an Ultrasonic Processor 500 W and 750 W (Sonics & Materials, Newtown, CT, USA) with a cycle of 15 s on and 3 s off and an amplitude of 30%. Unloaded MnWOx NPs were removed by centrifugation, and the pellet was washed three times with PBS.

Following the same procedure, T-DOX@MnWOx was prepared with the additional incorporation of DOX. Briefly, a 10 mg/mL solution of DOX in DMSO was prepared and subsequently added to the mixture consisting of 100 µL of 10% (*w*/*v*) T80 solution and 900 µL of PBS, followed by the addition of MnWOx and FAD dissolved in DCM. The formulation was then sonicated under the same conditions as used for MnWOx. The final product was purified by centrifuging and washing three times with PBS. The absorbance of DOX at 484 nm was measured to calculate the encapsulation efficiency of DOX [(amount of DOX loaded into T80)/(initial amount of DOX) × 100%] in various T80-based carriers.

The formulation optimization of MnWOx-loaded T80 nanocarriers was performed by varying the T80-to-FAD ratio while maintaining a constant amount of T80. The tested ratios included 10:0, 10:1, 10:2, 10:5, and 10:10. The same sonication and purification procedures were applied to all formulations. The preparation of FA-T-DOX@MnWOx was achieved by substituting FA-T80 for T80 in the aforementioned procedure.

### 3.3. Characterization of MnWOx NPs and MnWOx-Loaded T80-Based Carriers

For characterizing the chemical composition of the MnWOx NPs, EDS elemental mapping and XPS were adopted. The elemental composition of Mn and W in the MnWOx NPs was analyzed by inductively coupled plasma-optical emission spectrometry (ICP-OES, iCAP 7000, Thermo Fisher Scientific). DLS (Nano-ZS Zetasizer, Malvern, Worcestershire, UK) was used for the measurement of the hydrodynamic sizes and surface charges of various particles. The morphologies of various NPs were observed by TEM (Talos F200X, FEI, Hillsboro, OR, USA). A UV-Vis spectrophotometer (AquaMate 8100, Thermo Fisher Scientific) was used to acquire the absorbance spectra of various samples.

### 3.4. DOX Release Profiles from FA-T-DOX@MnWOx

The release profile of DOX from FA-T-DOX@MnWOx was assessed by incubating the particles in pH 7.4 PBS at 37 °C for 48 h. FA-T80 (0.5 mg) NPs containing DOX were dispersed in 100 μL of PBS and loaded into a dialysis device (Slide-A-Lyzer Mini Dialysis, MW cutoff = 20 kDa, Thermo Fisher Scientific). The amount of DOX released from FA-T-DOX@MnWOx was measured at 2, 4, 8, 12, and 24 h post-incubation. To investigate the drug release patterns of FA-T-DOX@MnWOx under US exposure, the samples were pre-treated with US (1 MHz, 0.5 W/cm^2^) for 1 min. The released amounts of DOX from FA-T-DOX@MnWOx were quantified by the absorbance of DOX, as previously reported [17].

### 3.5. In Vitro Study

U87MG cells were cultured in DMEM supplemented with 10% FBS and 1% antibiotics. Cellular uptake analysis, transcytosis study using an in vitro transwell BBB model [35], quantification of intracellular ROS and GSH, cytotoxicity evaluation [36], and apoptosis analysis were conducted. Detailed descriptions of each experiment are provided in the Supplementary Materials.

### 3.6. Statistical Analysis

Triplicate data, except where noted, are presented as mean ± SD. Statistical significance between groups was evaluated using one-way ANOVA, and the results are indicated as follows: * *p* < 0.05; ** *p* < 0.01.

## 4. Conclusions

We successfully developed cancer-targeted, MnWOx-loaded, T80-based nanocarriers for efficient BBB penetration and chemo-SDT against GBM. T80-based nanocarriers effectively co-encapsulated DOX (chemotherapeutic agent) and MnWOx (sonosensitizer). FA-T-DOX@MnWOx significantly promoted the BBB crossing of DOX. FA-T-DOX@MnWOx greatly enhanced the cellular uptake of DOX in U87MG cells. Notably, the FA conjugation of the T80-based nanocarriers further facilitated the cellular internalization of the DOX into GBM. In addition, FA-T-DOX@MnWOx triggered GSH depletion, synergistically enhancing intracellular ROS generation in U87MG cells upon US irradiation. As a result, FA-T-DOX@MnWOx triggered significant apoptosis in U87MG cells upon US irradiation. This study demonstrated that FA-conjugated, MnWOx-loaded, T80-based nanocarriers capable of crossing the BBB have great potential for the treatment of GBM via combined chemo-SDT. FA-conjugated T80-based nanocarriers are anticipated to be versatile BBB-crossing platforms for the effective loading of multiple anticancer drugs and nanoscale agents, enabling safe and efficient GBM therapy.

## Data Availability

The original contributions presented in this study are included in the article/Supplementary Materials. Further inquiries can be directed to the corresponding authors.

## Supplementary Materials

The following supporting information can be downloaded at: https://www.mdpi.com/article/10.3390/ijms262010235/s1.
