# Supplementary material for: Polysorbate-Based Carriers Encapsulating Oxygen-Deficient Nanoparticles for Targeted and Effective Chemo-Sonodynamic Therapy of Glioblastoma
Source: Int J Mol Sci. 2025 Oct 21;26(20):10235. doi: 10.3390/ijms262010235 (PMC12564566; doi:10.3390/ijms262010235)
Supplement: Supplementary file 1 [file ijms-26-10235-s001.zip › ijms-3900311-supplementary.pdf]

Supporting Information

Polysorbate-based carriers encapsulating oxygen-deficient nanoparticles for targeted and effective chemo-sonodynamic therapy of glioblastoma

Hyeon Ju Kang[†], Quan Truong Hoang[†], Nguyen Cao Nguyen, Binh Thi Thanh Pham, Thuy Giang Nguyen Cao, Vasanthan Ravichandran*, Min Suk Shim*

Department of Nano-Bioengineering, Incheon National University, Incheon 22012, Republic of Korea

[†] These authors contributed equally to this work.

*Corresponding authors:

Dr. Vasanthan Ravichandran, Email address: vasanthan.ravi@gmail.com

Dr. Min Suk Shim, E-mail address: msshim@inu.ac.kr

Experimental section

1. Characterization of MnWOx nanoparticles

X-ray photoelectron spectroscopy (XPS) was used to characterize chemical composition of MnWOx nanoparticles. XPS analysis was performed using a PHI Quantera SXM (ULVAC-PHI Inc., Tokyo, Japan) with an Al Kα monochromator source. The elemental composition of Mn and W in the MnWOx NPs were analyzed by inductively coupled plasma-optical emission spectrometry (ICP-OES, iCAP 7000, Thermo Fisher Scientific). The XPS spectra of MnWOx nanoparticles are represented Figure S1.

2. Synthesis of FA-conjugated Tween 80 (FA-T80)

2.1. Synthesis of Tween 80-succinate

Tween 80 (T80, 2.00 g, 1.52 mmol) was dissolved in 20 mL of dry CH_3CN, followed by the addition of succinic anhydride (0.41 g, 4.1 mmol) and 4-(dimethylamino)pyridine (DMAP, 18.3 mg, 0.15 mmol) (Sigma Aldrich) as a catalyst. The reaction mixture was refluxed at 80 °C overnight under stirring. After completion, the solvent was removed under reduced pressure, and the resulting oily residue was dissolved in $CHCl_3$ (100 mL). The organic solution was washed sequentially with 1 N HCl (3 × 40 mL) and deionized water (60 mL). The organic layer was dried over anhydrous $MgSO_4$, filtered, and concentrated under reduced pressure. The final product, Tween 80-succinate (Compound **1** in Figure S2) was obtained as a yellow oil in over 90% yield after solvent removal.

2.2. Synthesis of NHS ester-activated Tween 80 (T80-NHS ester)

Compound **1** (4.85 g, 3.44 mmol) was dissolved in $CHCl_3$ (20 mL) under nitrogen atmosphere. To this solution, *N*-hydroxysuccinimide (NHS, 0.43 g, 3.7 mmol) (Sigma Aldrich) and EDC·HCl (0.71 g, 3.7 mmol) (Sigma Aldrich) were added. The mixture was then stirred overnight at room temperature. After completion, the reaction mixture was diluted with CH_2Cl_2 (50 mL) and washed sequentially with 0.1 N HCl (2 × 40 mL) followed by brine (2 × 60 mL). The organic layer was dried over anhydrous $MgSO_4$, filtered, and concentrated under reduced pressure. The crude product was purified by silica gel column chromatography using a gradient of ethyl acetate/hexane (9:1), followed by $CHCl_3$/MeOH (9:1) as eluents. The T80-NHS ester

(Compound **2** in Figure S2) was obtained as a yellow oil in 89% yield.

2.3. Synthesis of folic acid-ethylenediamine conjugate (FA-EDA)

Folic acid (FA, 1.10 g, 2.5 mmol) (Sigma Aldrich) was dissolved in anhydrous dimethyl sulfoxide (DMSO) (20 mL) under a nitrogen atmosphere. HBTU (1.138 g, 3.0 mmol) and DIPEA (970 mg, 7.5 mmol) were added sequentially, and the mixture was stirred for 5 min to activate the carboxylic groups of FA. A solution of *N*-Boc-ethylenediamine (0.40 g, 2.5 mmol) (Sigma Aldrich) in DMSO (5 mL) was then added dropwise to the above mixture at room temperature. The reaction was continued for 18 h at room temperature. Upon completion, the reaction mixture was poured into cold water to induce precipitation. The resulting precipitate was collected by centrifugation and washed with cold water three times to remove excess reagents and byproducts. The purified product was lyophilized to afford the *N*-Boc-protected FA derivative (Compound **3** in Figure S2) as a yellow powder.

Compound **3** (1.00 g, 1.71 mmol) was then treated with trifluoroacetic acid (TFA, 8 mL) (Sigma Aldrich) at room temperature. The reaction mixture was stirred for 3 h, after which the TFA was removed under high vacuum using a rotary evaporator. The resulting greasy residue was washed and precipitated with cold diethyl ether to afford the FA-ethylenediamine conjugate (FA-EDA) as a solid (Compound **4** in Figure S2).

2.4. Synthesis of FA-conjugated Tween 80 (FA-T80)

T80-NHS ester (Compound **2**) (500 mg, ~0.33 mmol) was dissolved in 2 mL of dry *N*,*N*-dimethylformamide (DMF), followed by the addition of DIPEA (122 μL, 0.70 mmol) and FA-EDA (Compound **4**, 0.24 g, 0.49 mmol). The reaction mixture was stirred at room temperature for 24 h. Upon completion, the product was precipitated with cold diethyl ether. The resulting solid was collected and lyophilized and stored at –4 °C.

3. Synthesis of lipid-like fatty amine derivative (FAD)

Lipid-like fatty amine derivative (FAD) was synthesized following the same method in a previous report (Figure S6) [34]. Briefly, 120 mg of adipic acid (0.82 mmol, 1 equiv.) (Sigma Aldrich) was dissolved into $CHCl_3$ (5 mL). After the adipic acid had dissolved completely, it was mixed with *N*,*N*-diisopropylethylamine (DIPEA, 635 mg, 4.92 mmol, 6 equiv.) (Sigma

Aldrich) and *N,N,N′,N′*-Tetramethyl-O-(1H-benzotriazol-1-yl)uronium hexafluorophosphate (HBTU, 2.70 mmol, 1.03 g, 3.3 equiv.) (Sigma Aldrich). After 5 min of stirring, 722 mg of oleylamine (2.70 mmol, 3.3 equiv.) was added dropwise to the reaction solution. After 12 h of stirring at room temperature, the solvent was evaporated. The resulting crude product was extracted with $CHCl_3$ after washing with HCl aqueous solution. Purification was achieved using silica gel chromatography with a gradient elution from 4:1 to 1:1 hexane:ethyl acetate, yielding a yellow solid product.

4. Cellular uptake analysis

b.End3 and U87MG cells were prepared for the assessment of cellular uptake of FTAD@MnWOx. bEnd.3 and U87MG cells were cultured in 10% FBS-containing DMEM. b.End3 and U87MG cells were seeded in 6-well plates at the density of 2.5×10^5 cells per well, 24 h before the treatment. Subsequently, the cells were exposed to free DOX, T@MnWOx, and FA-T-DOX@MnWOx (2.5 μg/mL DOX) for 4 h. Following trypsinization to collect the cells, the red fluorescence emitted from DOX within the cells was quantified using a flow cytometer (CytoFLEX, Beckman Coulter, Brea, CA, USA).

To investigate the targeting ability of FA-T-DOX@MnWOx toward glioblastoma compared to normal brain endothelial cells, the uptake levels of T-DOX@MnWOx and FA-T-DOX@MnWOx were assessed in b.End3 and U87MG cells. b.End3 and U87MG cells were seeded at a density of 2.5×10^5 cells per well in 24-well plates. After 24 h of incubation, the cells were exposed to T-DOX@MnWOx and FA-T-DOX@MnWOx for 4 h. Subsequently, the cells were harvested via trypsinization, and the red fluorescence of DOX in the cells was quantified using by flow cytometry (CytoFLEX).

To examine the transcytosis mechanism of FA-T-DOX@MnWOx through folate receptors, U87MG cells were cultured in 12-wellplate (2.5×10^5 cells/well). After 24 h of incubation, the cells were washed with PBS. Subsequently, the cells were pre-treated with FA at a concentration of 10 mM for 1 h. After washing with PBS, the cells were incubated with TAD@MnWOx, FTAD@MnWOx for 4 h at the same concentration of DOX and MnWOx. The cells that were not pre-treated with FA were also incubated with T-DOX@MnWOx and FA-T-DOX@MnWOx as a control. Following trysinization, the fluorescence of DOX in the cells was measured using flow cytometry (CytoFLEX).

5. Transcytosis study using an in vitro transwell BBB model

BBB permeability of various samples (free DOX, T-DOX@MnWOx, and FA-T-DOX@MnWOx) was determined using an in vitro transwell BBB model, as previously reported [35]. Briefly, 400 µL of cell culture media of bEnd.3 cells (5×10^6 cells per well) were seeded on the upper side of transwell inserts (0.4 µm pore size, Corning-Costar Corp., Corning, NY). U87MG cells (5×10^6 cells/well) were seeded in the lower chamber with 800 µL of culture media. The culture media were replaced every other day. The integrity of cell layers was verified by measuring trans-epithelial electrical resistance (TEER) using an EVOM2 epithelial voltohmeter (World Precision Instruments Inc., Sarasota, FL, USA). The multilayered cells were cultured until their TEER values exceeded 200 $\Omega \cdot cm^2$. After discarding the medium in the upper compartment, the FBS-free media containing various samples (DOX: 2.5 µg/mL, MnWOx: 7.5 µg/mL) was added into the compartment and incubated for 4 h. The amount of DOX that crossed the cell layer was then quantified by measuring the absorbance at 482 nm using a UV-Vis spectrophotometer (SpectraMax® iD3). The apparent permeability coefficients (P_{app}) of T-DOX@MnWOx and FA-T-DOX@MnWOx were calculated using the following formula:

$$P_{app} = \frac{C_{receiver} \times V_{receiver}}{A \times \ t \times C_{donor\ initial}}$$

where A is membrane area (0.33 cm^2); t is incubation time (4 h); $C_{donor\ intitial}$ is initial concentration of donor (upper compartment, µg/mL); $C_{receiver}$ is concentration of receiver (lower compartment, µg/mL); $V_{receiver}$ is volume of receiver (lower compartment, cm^3).

To evaluate the internalization of T-DOX@MnWOx, and FA-T-DOX@MnWOx into U87MG human glioblastoma cells after their transcytosis of brain endothelial cells, the fluorescence of DOX in U87MG cells cultured in lower side were quantified using a flow cytometer.

6. Quantification of intracellular ROS and GSH

Intracellular ROS generation in U87MG cells after various treatments upon US irradiation was quantified using 2′,7′-dichlorofluorescein diacetate (DCF-DA). The cells were stained with

10 μM DCF-DA and then treated with free DOX, T@MnWOx, T-DOX@MnWOx, and FA-T-DOX@MnWOx at a concentration of 2.5 μg/mL DOX and 7.5 μg/mL MnWOx (370 μg/mL T80). After 4 h of incubation, the cells were washed with PBS. Then, the bottom of the well plate with a layer of cold US gel was treated with 0.5 W/cm^2 for 2 min (1 MHz). After 30 min of incubation, the cells were trypsinized and re-suspended in PBS. The green fluorescence in the cells indicating intracellular ROS was quantitatively analyzed using a flow cytometer (CytoFLEX). Fluorescence imaging was also performed using a fluorescence microscope (Nikon Eclipse Ti-S, Nikon, Tokyo, Japan).

To quantify intracellular GSH levels, U87MG cells were seeded into 12-well plates at a density of 1 × 10^5 cells per well and kept overnight. Afterward, the medium was replaced with a fresh medium containing various formulations of free DOX, T@MnWOx, T-DOX@MnWOx, and FA-T-DOX@MnWOx. After 4 h of treatment, the GSH content in the cells was quantified using a GSH assay kit (DoGenBio, Seoul, South Korea) in accordance with the manufacturer's instructions.

7. In vitro cytotoxicity evaluation

The cytotoxicity of various samples against U87MG cells was evaluated using a conventional MTT assay. 96-well plates were seeded with U87MG cells (1 × 10^4 cells/well) and incubated for 24 h. Then, the cells were treated with free DOX, T@MnWOx, T-DOX@MnWOx, FA-T-DOX@MnWOx at a concentration of 2.5 μg/mL DOX and 7.5 μg/mL MnWOx (370 μg/mL T80), followed by co-incubation for 4 h. Untreated cells were used as a control. After 4 h of incubation, US was applied to the bottom of the well with a cold US gel layer for 2 min at 0.5 W/cm^2. Then, the medium in the well was replaced with a fresh culture medium and incubated for 24 h. The viabilities of the cells were measured using an MTT powder, as previously reported [36].

8. Apoptosis analysis

U87MG cells were seeded into 12-well plates at a density of 3 × 10^5 cells per well and incubated for 24 h before the treatment. The cells were treated free DOX, T@MnWOx, T-DOX@MnWOx, and FA-T-DOX@MnWOx (2.5 μg/mL DOX, 7.5 μg/mL MnWOx), followed by incubation for 4 h. Then the medium in the well was replaced with fresh culture medium.

After a cold US gel was applied under the well plate, US (was exposed to the cells for 2 min. The cells were cultured for an additional 24 h. Annexin V-FITC and PI staining was carried out following the manufacturer’s protocol (BD Biosciences, San Jose, CA, USA), as previously reported [36].

Table S1. Sizes and zeta potentials of various T-DOX@MnWOx nanocarriers with varying T80-to-FAD ratios

Samples	T80-to-FAD ratio (w/w)	Size (nm)	Zeta potential (mV)
T-DOX@MnWOx	10:0	870.4 ± 81.3	2.32 ± 0.42
	10:1	433.2 ± 38.9	3.40 ± 0.28
	10:2	214.6 ± 3.2	5.82 ± 1.09
	10:5	382.7 ± 45.3	1.41 ± 0.36
	10:10	459.4 ± 41.3	–0.29 ± 0.30

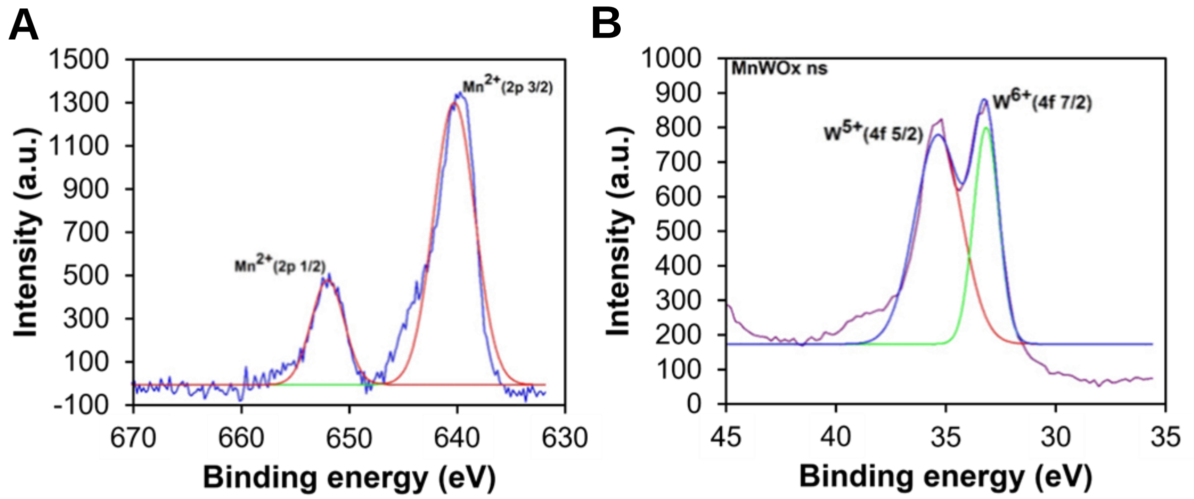

Figure S1. High-resolution XPS spectra of MnWOx nanoparticles.

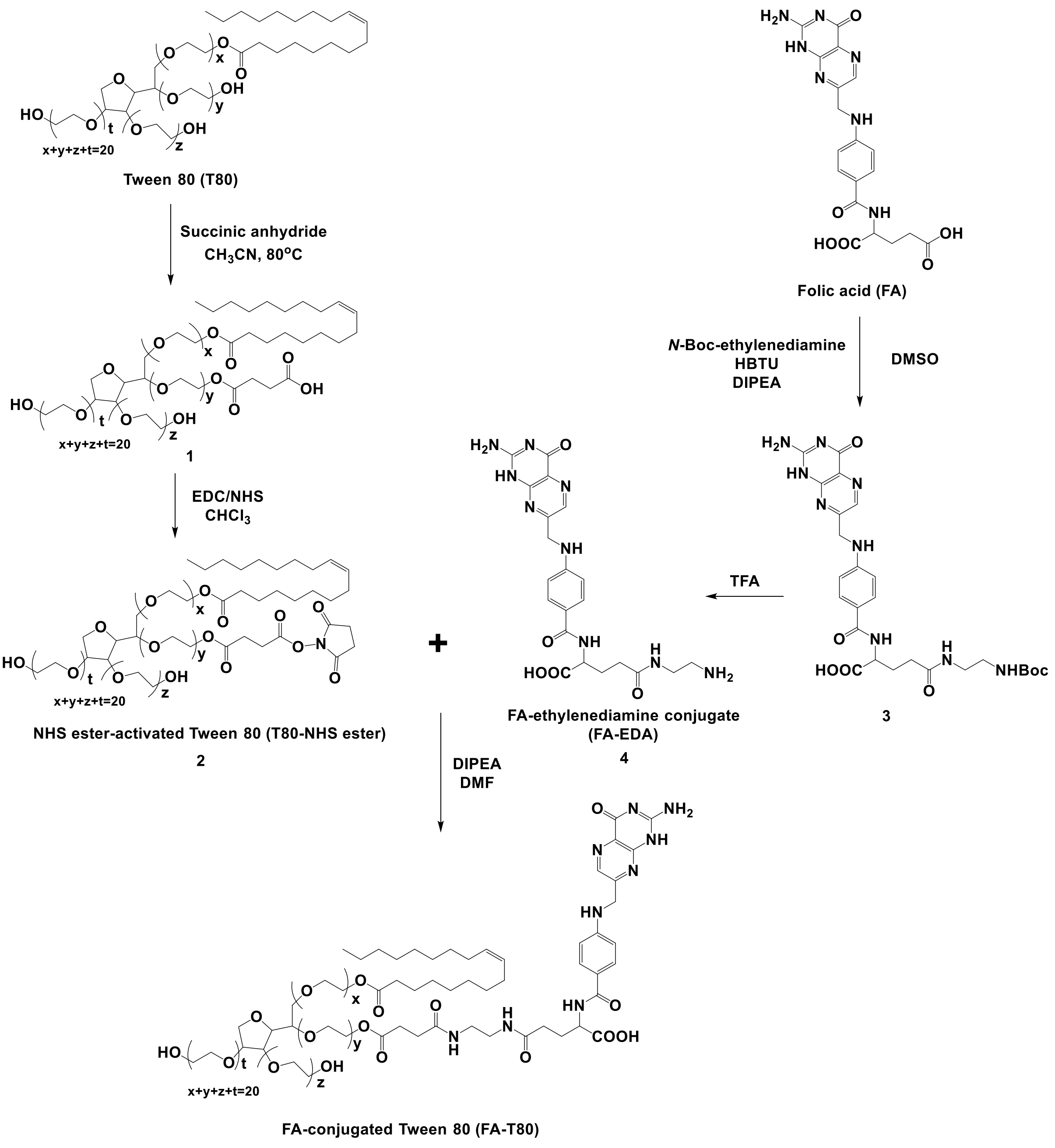

Figure S2. Synthetic scheme for the preparation of FA-conjugated Tween 80 (FA-T80).

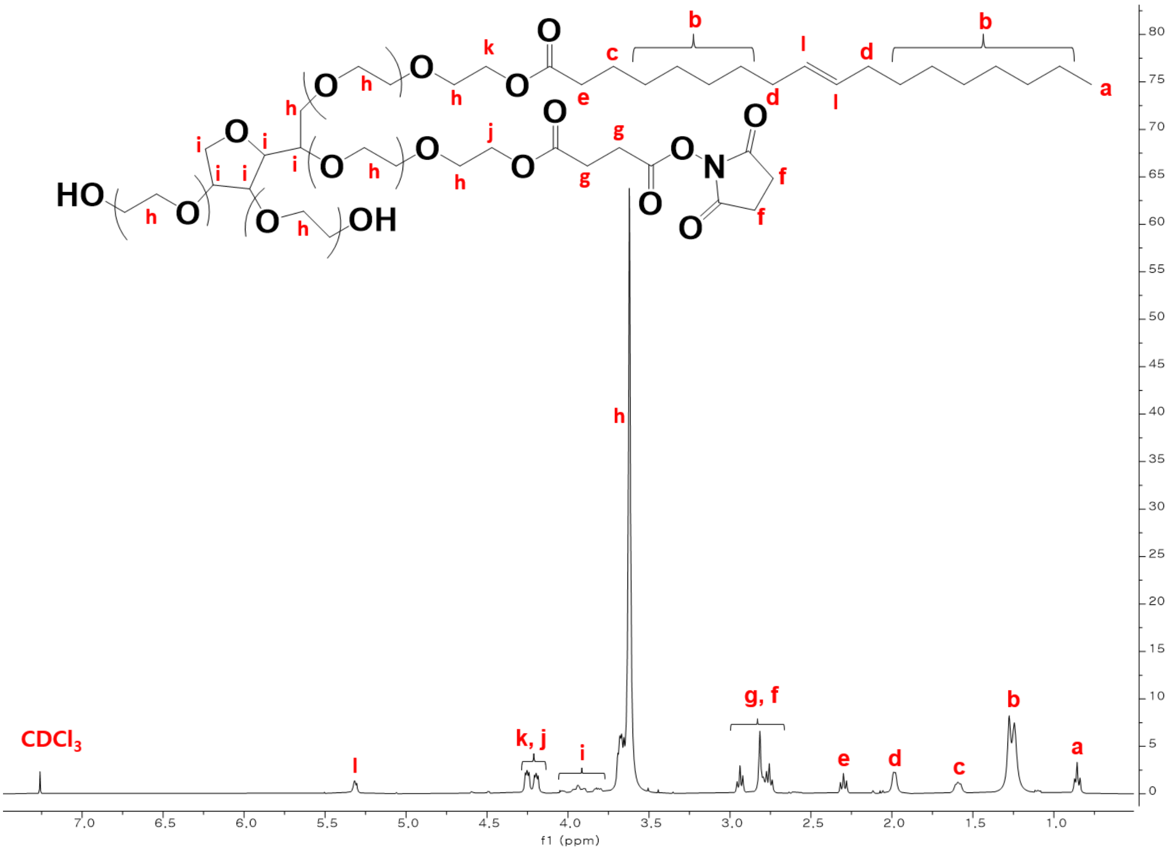

Figure S3. ^{1}H NMR spectra of (A) Tween 80-succinate (Compound **1** in Figure S2) and (B) T80-NHS ester (Compound **2** in Figure S2).

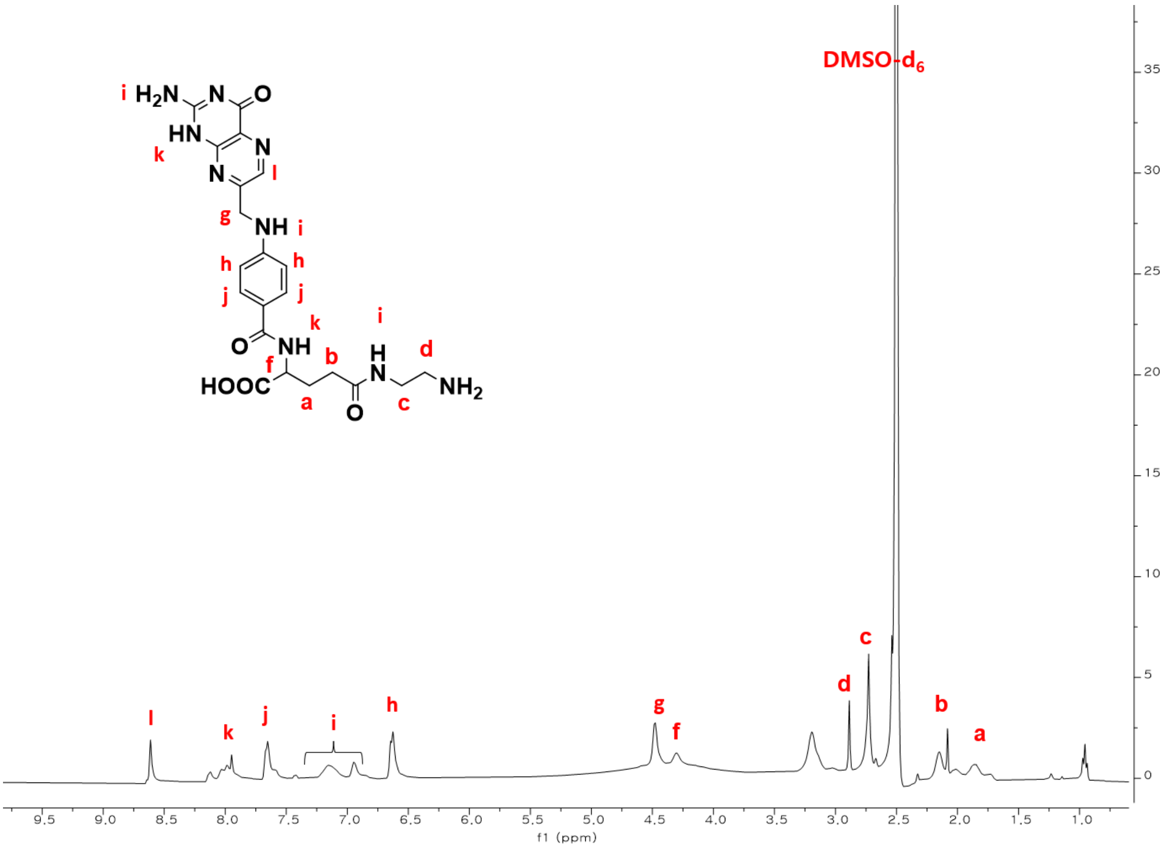

Figure S4. ^{1}H NMR spectrum of FA-EDA.

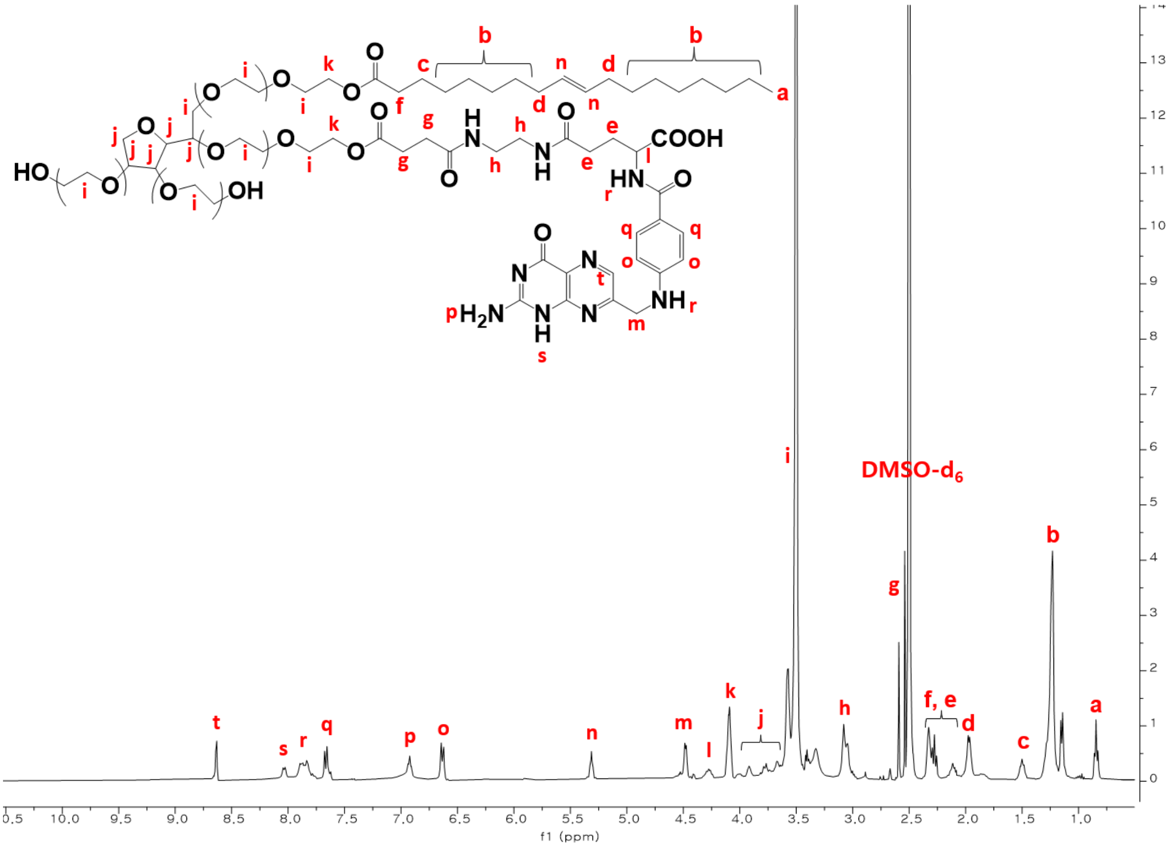

Figure S5. ^{1}H NMR spectrum of FA-conjugated Tween 80 (FA-T80)

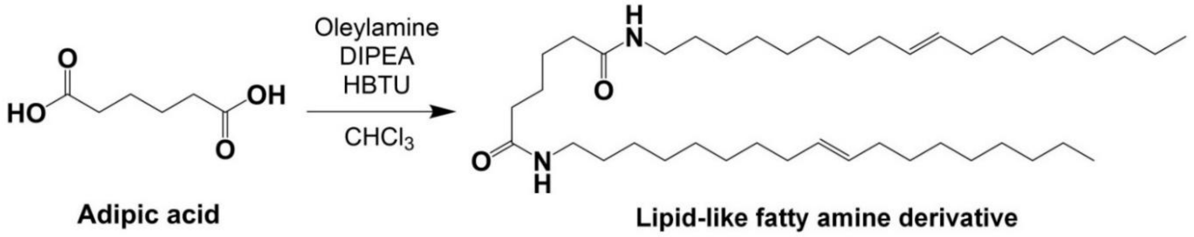

Figure S6. Synthetic scheme for the synthesis of lipid-like fatty amine derivative (FAD).

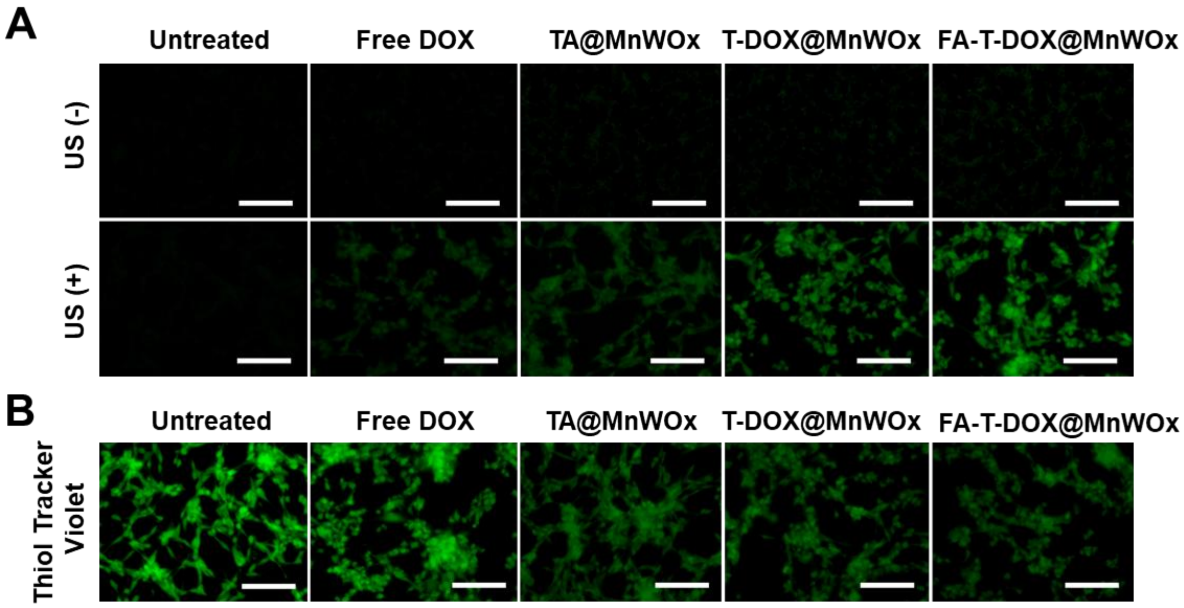

Figure S7. (A) Fluorescence images of intracellular ROS in U87MG cells treated with various samples before and after US irradiation (0.5 W/cm^2, 1 MHz, 2 min). (B) Fluorescence images of intracellular GSH level in U87MG cells treated with various samples. Scale bar = 100 μm.
